# Nutrient removal by rice–wheat cropping system as influenced by crop establishment techniques and fertilization options in conjunction with microbial inoculation

**DOI:** 10.1038/s41598-020-78729-w

**Published:** 2020-12-15

**Authors:** Amit Anil Shahane, Yashbir Singh Shivay, Radha Prasanna, Dinesh Kumar

**Affiliations:** 1 Division of Agronomy, ICAR-Indian Agricultural Research Institute, New Delhi, 110 012 India; 2 Division of Microbiology, ICAR-Indian Agricultural Research Institute, New Delhi, 110 012 India

**Keywords:** Microbiology, Plant sciences

## Abstract

Nutrient uptake by the rice–wheat cropping system (RWCS) is an important indicator of soil fertility and plant nutrient status. The hypothesis of this investigation was that the rate and sources of nutrient application can differentially influence nutrient removal and soil nutrient status in different crop establishment techniques (CETs). Cropping system yield was on par in all the CETs evaluated, however, there were significant changes in soil nutrient availability and microbiological aspects. The system nitrogen (N), phosphorus (P), potassium (K) and zinc (Zn) uptake in aerobic rice system followed by zero tillage wheat (ARS-ZTW) was 15.7–17.6 kg ha^−1^, 0.7–0.9 kg ha^−1^, 7–9.8 kg ha^−1^ and 13.5–23.1 g ha^−1^ and higher than other CETs. The formulations of *Anabaena* sp. (CR1) + *Providencia* sp. (PR3) consortium (MC1) and *Anabaena*–*Pseudomonas* biofilm (MC2) recorded significantly higher values of soil chlorophyll and microbial biomass carbon and positively affected cropping system nutrient uptake and soil nutrient balance, illustrating the beneficial effect of microbial inoculation through increased supply of biologically fixed N and solubilised P. Zinc fertilization (5 kg Zn ha^−1^ through ZnSO_4_·7H_2_O as soil application) increased soil DTPA-extractable Zn by 4.025–4.836 g ha^–1^, with enhancement to the tune of 20–24% after two cropping cycles of RWCS. Our investigation recommends the need for change in the present CETs to ARS–ZTW, along with the use of microbial inoculation as a means of significantly enhancing cropping system nutrient uptake and soil nutrient status improvement.

## Introduction

In the present day scenario, changes in investigations on crop establishment techniques (CETs) and management practices in rice and wheat are getting more emphasis^1–3^. This is mainly because of variations in CETs with respect to their resource utilization^4^, energy requirements^5,6^, capacity to act as a mitigation strategy for climate change^7^ can have far reaching implications in terms of yield and income to the farmers^8,9^, besides environmental health. Additionally, the adoption of new CETs and management practices are becoming increasingly significant to address the issues related to degradation of natural resources and increasing cost of chemical and agronomic interventions or resources.

Among the different CETs of rice, the system of rice intensification (SRI) is one which was invented in Madagaskar Island by the Father Hendra De Laolani in 1983 and promoted by researchers^10,11^ over different part of the world. The SRI is getting momentum despite of different opinion about its superiority in term of yield^12,13^. In some places, only one or few components/recommendations of SRI are followed (modified SRI) which was found to be economically suitable^14^. Its adaptation is mostly promoted for its higher water productivity even though grain yield may remain same as that of conventional puddled transplanted rice^15^. The superiority of SRI in yield potential was not consistent over different part of the world and report of higher, lower as well as similar yield over conventional transplanted rice are accumulated and reviewed by several authors^16–18^. Another CET getting attention mainly due to its immense water saving potential is aerobic rice system (ARS)^19^. The eastern and north-eastern states of India grow rice as rainfed upland crop on nearly 6 million ha area^20^. The lower yield level^21^, higher weed infestation and cost on weed management^22^, iron deficiency^23^ and problems of nematodes^24^ are important issues with ARS which need to be addressed to make it a potential alternative to puddled transplanted rice (PTR).

In India about 10 million ha area under rice cultivation is planted with wheat after rice harvest^25^. The soil condition and residual effects of previous season rice crop affect the performance of succeeding wheat. The short turn-around period (duration between harvesting of rice and sowing of wheat) and disposal of rice residue in rice–wheat cropping system (RWCS) leads to increase in adaptation of zero tillage planting of wheat^8^ in India. The zero tillage planting was also promoted for its energy efficiency^26^. The availability of sowing machine for small land holdings, variable options for residue management, change in seed rate and nitrogen fertilization, as well as increased use of herbicide for weed management have facilitated efficient wheat agronomy, after the introduction of zero tillage wheat planting. Along with zero tillage, another CET followed and getting attention is the system of wheat intensification^27^ which is the application of SRI principles to wheat.

These CETs have varied levels of impact on soil properties, plants performance and nutrient and water availability^3,28,29^. Such impacts can act as a guide for modulating the recommendations and input portfolio of crop and/or cropping system. The rice and wheat crops together consume nitrogen to the tune of 7.9 million tonnes (mt) (52.5% of the total nitrogen used in India), 3.42 mt (48.4% of the total P_2_O_5_ used in India) of P_2_O_5_ and 1.2 mt (46.7% of the total K_2_O used in India) K_2_O through fertilizer^30^. The energy consumption in rice and wheat production in India is 572.5 × 10^9^ MJ and 433.5 × 10^9^ MJ, respectively; while rice alone consumes 18% of total agrochemicals used in India^31^. The contribution of these two crops to food grain and cereal production in India is 74.8% and 80.9%^32^. Considering the significant contribution of these two crops to the resource consumption and production, a detailed study of the effects of CETs in RWCS is vital, particularly in relation to nutrient dynamics.

The present status of soil nutrient balance in India is negative (10 million tonnes)^33^, which is the result of two important considerations. The first one relates to the increasing nutrient uptake due to round the year cropping to fulfil the needs of increasing human and cattle population; while the other represents the finite nutrient reserve, low nutrient addition as compared to removal and imbalanced nutrient application practices. In this context, a comparative study of CETs for their nutrient uptake is of prime importance.

In the present study, six CETs were studied for their potential to influence nitrogen (N), phosphorus (P), potassium (K), zinc (Zn) and iron (Fe) uptake in RWCS, soil microbial parameters and soil P (NaHCO_3_-extractable fraction), K (1 N ammonium acetate-extractable fraction) and Zn (DTPA-extractable fraction) after completion of first and second cropping cycle of RWCS. Along with CETs, rate of N and P nutrient application through chemical fertilizer, Zn fertilization and microbial inoculation are the other variables studied in the present investigation.

The chemical fertilizers have their monopoly among sources of nutrient inputs used in RWCS. The artificial nature of chemical fertilizers, costly and high energy demanding processes involved in their production and emerging need for reduction in their monopoly are the important concerns of use of chemical fertilizers. These concerns are addressed partly through identification, quantification and utilization of location-specific as well as worldwide applicable complementary and supplementary viable options of crop nutrition^34–36^. The present study utilizes the complementary options of application of microbial consortia of N fixing and P solubilising microorganisms for nitrogen and phosphorus nutrition of RWCS. The performance of these microbial consortia were studied in different water regimes (as in case of rice) and varied residual effect and tillage (as in case of wheat). The significance of use of N fixing microorganisms^34,37^ in present day agriculture is particularly justified by different factors such as the adverse effect of excessive use of nitrogenous fertilizers on ecosystem health, energy consumption in the process of fertilizer preparation and increased need of proteins (which need higher N fertilization) due to over increasing human and domestic animal population. Along with N fixing microorganisms, use of P solubilising and mobilizing microorganisms^38^ also need to be emphasized as P use efficiency is only 15–20%, with remaining P get fixed in soil. The share of this fixed P in crop nutrition can be increased by the use of these microorganisms.

The justification for selection of Zn fertilization as a treatment was based on three types of Zn deficiencies. Among them, the first one is soil Zn deficiency. Out of 0.251 million samples analyzed from different part of India, 49% of samples were found deficient in zinc^39^. The second type of deficiency is related to the plant response in terms of yield enhancement^40^ and nutrient enrichment of crop^41^; while third type of deficiency is directly related with human nutrition^42^. With this background, the study was planned to get insight in to significance of CETs and rate and sources of fertilization on nutrient uptake and soil nutrient status in RWCS.

## Results

### Biological yield of the cropping system

The biological yield of the cropping system was not affected significantly due to CETs (Table 1); while nutrient management treatments differed significantly. Application of RDN + Zn in ARS–ZTW recorded the highest biological yield which remained on par with 75% RDN + MC1 + Zn and 75% RDN + MC2 + Zn in all three CETs of RWCS. Application of MC1 and MC2 increased the cropping system biological yield by 0.99–1.11 Mg ha^−1^ and 1.12–1.19 Mg ha^−1^, respectively. Zn fertilization increased the cropping system biological yield by 0.77–1.06, 0.36–0.46, 0.91–1.07 and 0.88–0.95 Mg ha^−1^ , when applied along with RDN, 75% RDN, 75% RDN + MC1 and 75% RDN + MC2, respectively.

**Table 1:** Influence of crop establishment techniques and nutrient management options on biological yield (Mg ha^−1^) of rice–wheat cropping system during 2013–2014 and 2014–2015.

Treatment	Control	RDN	RDN* + Zn**	75% RDN	75% RDN + Zn	75% RDN + MC1	75% RDN + MC1 + Zn	75% RDN + MC2	75% RDN + MC2 + Zn	Mean
2013–2014										
PTR-CDW	18.75	22.32	23.24	20.92	21.34	22.03	22.94	22.11	22.96	21.85
SRI-SWI	19.15	22.31	22.81	20.88	21.38	21.95	22.87	22.06	22.92	21.82
ARS-ZTW	19.14	22.46	23.35	21.02	21.48	22.16	23.05	22.23	23.15	22.01
Mean	19.02	22.37	23.14	20.94	21.40	22.05	22.96	22.13	23.01	

	Crop establishment techniques	Nutrient management options	Interaction							
SEm ±	0.05	0.12	0.21							
CD (*p* = 0.05)	0.20	0.35	NS							

2014–2015										
PTR–CDW	17.90	22.13	23.21	20.89	21.18	21.92	22.99	22.05	22.93	21.69
SRI–SWI	18.69	22.00	23.05	20.76	21.14	21.74	22.80	21.87	22.85	21.66
ARS–ZTW	18.84	22.17	23.21	20.93	21.32	21.90	22.97	22.03	23.01	21.82
Mean	18.48	22.10	23.16	20.86	21.22	21.85	22.92	21.98	22.93	

	Crop establishment techniques	Nutrient management options	Interaction							
SEm ±	0.07	0.15	0.26							
CD (*p* = 0.05)	0.29	0.43	0.74							

*PTR*, puddled transplanted rice; *SRI*, system of rice intensification; *ARS*, aerobic rice system; *CDW*, conventional drill-sown wheat; *SWI*, system of wheat intensification, *ZTW*, zero tillage wheat; RDN*, recommended dose of nutrients [120 kg nitrogen ha^−1^ and 25.8 kg phosphorus (P) ha^−1^ per crop]; Zn**, 5 kg Zn ha^−1^ through ZnSO_4_·7H_2_O per crop, *MC1*, *Anabaena* sp. (CR1) + *Providencia* sp. (PR3) consortium; *MC2*, *Anabaena*–*Pseudomonas* biofilm; Potassium (K) was applied uniformly in all treatments @ 49.8 kg K ha^−1^ per crop; *Interaction*, non-significant in 2013–2014 and significant in 2014–2015.

### Cropping system related N, P and K uptake

The cropping system nitrogen uptake varied from 129.4 to 290.2 kg ha^−1^ with the highest in ARS–ZTW (237.7–245.7 kg ha^−1^) among CETs and RDN + Zn (281–290 kg ha^−1^) within nutrient management treatments (Table 2). The application of microbial inoculation increased system N uptake by 28.3 to 33.0 kg ha^−1^. Zinc fertilization increased the cropping system N uptake by 34.5, 6.3, 33.9 and 36.0 kg ha^−1^ when applied along with RDN, 75% RDN, 75% RDN + MC1 + Zn and 75% RDN + MC2 + Zn, respectively in the first year and similar results were also recorded in the 2nd year. This showed that, application of Zn with 75% RDN + MC2 was better than the application with RDN. The increase in cropping system N uptake due to application of RDN was 38.3–39.7 kg ha^−1^ and 89.7–94.5 kg ha^−1^ over 75% RDN and control, respectively.

**Table 2:** Influence of crop establishment techniques and nutrient management options on nitrogen uptake (kg ha^−1^) in rice–wheat cropping system during 2013–2014 and 2014–2015.

Treatment	Control	RDN	RDN* + Zn**	75% RDN	75% RDN + Zn	75% RDN + MC1	75% RDN + MC1 + Zn	75% RDN + MC2	75% RDN + MC2 + Zn	Mean
2013–2014										
PTR-CDW	145.5	241.4	276.1	200.0	205.7	232.9	263.8	233.9	268.6	229.8
SRI-SWI	152.2	241.4	273.3	200.2	207.1	231.4	263.3	233.4	267.5	230.0
ARS-ZTW	169.0	253.1	290.2	216.5	223.2	245.3	284.1	245.3	284.5	245.7
Mean	155.6	245.3	279.8	205.6	211.9	236.5	270.4	237.5	273.5	

	Crop establishment techniques	Nutrient management options	Interaction							
SEm±	1.56	3.88	6.72							
CD (*p* = 0.05)	6.12	11.03	19.10							

2014–2015										
PTR-CDW	129.4	231.6	266.5	191.4	196.3	221.8	256.3	226.6	260.7	220.1
SRI-SWI	137.5	230.7	265.5	191.0	197.1	219.6	255.1	225.1	259.3	220.1
ARS-ZTW	156.6	244.8	281.8	209.7	216.1	235.7	277.3	239.5	278.0	237.7
Mean	141.2	235.7	271.2	197.4	203.1	225.7	262.9	230.4	266.0	

	Crop establishment techniques	Nutrient management options	Interaction							
SEm±	1.48	3.57	6.18							
CD (*p* = 0.05)	5.83	10.15	17.57							

*PTR*, puddled transplanted rice; *SRI*, system of rice intensification; *ARS*, aerobic rice system; *CDW*, conventional drill-sown wheat; *SWI*, system of wheat intensification; *ZTW*, zero tillage wheat; RDN*, recommended dose of nutrients [120 kg nitrogen ha^−1^ and 25.8 kg phosphorus (P) ha^−1^ per crop]; Zn**, 5 kg Zn ha^−1^ through ZnSO_4_·7H_2_O per crop; *MC1*, *Anabaena sp.* (CR1) + *Providencia sp.* (PR3) consortium; *MC2*, *Anabaena*–*Pseudomonas* biofilm; Potassium (K) was applied uniformly in all treatments @ 49.8 kg K ha^–1^ per crop; *Interaction*, significant in both cropping cycle.

In case of P, application of 75% RDN with MC1 or MC2 + Zn in ARS–ZTW had 5.4–6.2% and 6.5–6.9% higher P uptake than same treatment applied in PTR–CDW and SRI–SWI (Table 3). Similarly, for K, this increase was 21.9–26.5 and 25.4–29.1 kg ha^−1^ even though K application rate was remained same in all CETs and nutrient management treatments (Table 4). The increase in P uptake due to application of RDN + Zn in ARS–ZTW was 0.8–1.0 and 0.7–1.0 kg ha^−1^ over the same treatment applied in PTR–CDW and SRI–SWI, respectively; while for K it was 6.8–7.8 and 9.4–12 kg ha^−1^. The overall effect of this treatment superiority was reflected in significantly higher P and K uptake in ARS–ZTW.

**Table 3:** Influence of crop establishment techniques and nutrient management options on phosphorus uptake (kg P ha^−1^) in rice–wheat cropping system during 2013–2014 and 2014–2015.

Treatment	Control	RDN	RDN* + Zn**	75% RDN	75% RDN + Zn	75% RDN + MC1	75% RDN + MC1 + Zn	75% RDN + MC2	75% RDN + MC2 + Zn	Mean
2013–2014										
PTR-CDW	21.6	27.9	28.8	25.6	26.2	27.0	28.4	27.3	28.9	26.9
SRI-SWI	22.4	27.8	28.8	25.7	26.1	26.9	28.7	27.5	28.3	26.9
ARS-ZTW	23.2	28.4	29.8	26.2	26.8	28.4	29.4	28.1	29.6	27.8
Mean	22.4	28.0	29.1	25.8	26.4	27.4	28.8	27.6	28.9	

	Crop establishment techniques	Nutrient management options	Interaction							
SEm±	0.10	0.15	0.25							
CD (*p* = 0.05)	0.38	0.42	0.72							

**2014–2015**										
PTR-CDW	20.8	27.6	28.6	25.6	26.1	26.9	28.3	27.2	28.6	26.6
SRI-SWI	21.8	27.3	28.7	25.6	26.0	26.7	28.7	27.3	28.0	26.7
ARS-ZTW	22.6	27.9	29.4	26.1	26.6	28.2	28.7	27.9	29.2	27.4
Mean	21.7	27.6	28.9	25.8	26.2	27.2	28.5	27.5	28.6	

	Crop establishment techniques	Nutrient management options	Interaction							
SEm±	0.11	0.16	0.29							
CD (*p* = 0.05)	0.41	0.47	0.81							

*PTR*, puddled transplanted rice; *SRI*, system of rice intensification; *ARS*, aerobic rice system; *CDW*, conventional drill-sown wheat; *SWI*, system of wheat intensification; *ZTW*, zero tillage wheat; RDN*, recommended dose of nutrients [120 kg nitrogen ha^−1^ and 25.8 kg phosphorus (P) ha^−1^ per crop]; Zn**, 5 kg Zn ha^−1^ through ZnSO_4_·7H_2_O per crop; *MC1*, *Anabaena* sp*.* (CR1) + *Providencia* sp*.* (PR3) consortium; *MC2*, *Anabaena*–*Pseudomonas* biofilm; Potassium (K) was applied uniformly in all treatments @ 49.8 kg K ha^−1^ per crop; *Interaction*, significant in both cropping cycle.

**Table 4:** Influence of crop establishment techniques and nutrient management options on potassium uptake (kg K ha^−1^) in rice–wheat cropping system during 2013–2014 and 2014–2015.

Treatment	Control	RDN	RDN* + Zn**	75% RDN	75% RDN + Zn	75% RDN + MC1	75% RDN + MC1 + Zn	75% RDN + MC2	75% RDN + MC2 + Zn	Mean
2013–2014										
PTR-CDW	152.3	238.4	250.6	203.9	211.4	231.5	244.1	234.0	248.1	223.8
SRI-SWI	155.7	237.6	245.4	204.4	212.0	230.5	244.0	233.4	246.5	223.3
ARS-ZTW	161.0	244.0	257.4	211.7	218.9	237.6	250.9	239.9	255.7	230.8
Mean	156.3	239.9	251.1	206.7	214.1	233.2	246.5	235.8	250.0	

	Crop establishment techniques	Nutrient management options	Interaction							
SEm±	0.46	2.61	4.53							
CD (*p* = 0.05)	1.80	7.43	12.87							

2014–2015										
PTR-CDW	141.0	229.9	243.2	200.5	204.3	224.0	239.9	227.6	240.2	216.7
SRI-SWI	148.6	227.3	241.6	199.7	203.6	221.4	238.0	225.0	237.9	215.9
ARS-ZTW	158.1	236.9	251.0	210.2	214.5	230.8	247.0	234.3	248.8	225.7
Mean	149.2	231.3	245.3	203.5	207.5	225.4	241.6	228.9	242.3	

	Crop establishment techniques	Nutrient management options	Interaction							
SEm±	0.65	2.59	4.49							
CD (*p* = 0.05)	2.55	7.38	12.78							

*PTR*, puddled transplanted rice; *SRI*, system of rice intensification; *ARS*, aerobic rice system; *CDW*, conventional drill-sown wheat; *SWI*, system of wheat intensification; *ZTW*, zero tillage wheat; RDN*, recommended dose of nutrients [120 kg nitrogen ha^−1^ and 25.8 kg phosphorus (P) ha^−1^ per crop]; Zn**, 5 kg Zn ha^−1^ through ZnSO_4_·7H_2_O per crop; *MC1*, *Anabaena* sp*.* (CR1) + *Providencia* sp*.* (PR3) consortium; *MC2*, *Anabaena*–*Pseudomonas* biofilm; Potassium (K) was applied uniformly in all treatments @ 49.8 kg K ha^−1^ per crop; *Interaction*, non-significant in 2013–2014 and significant in 2014–2015.

### Cropping system related Zn and Fe uptake

Among the treatment variables analysed, the highest enhancement in Zn uptake was recorded with rate of N and P application followed by microbial inoculation (Table 5). The increase in Zn uptake in RWCS due to rate of N and P application, microbial inoculation, Zn fertilization and CETs were 101.4–282.7, 88.3–95.5, 76.8–79.3 and 18.3–23.1 g ha^−1^, respectively. In case of Fe uptake, the rate of N and P application (457.8–1350.6 g ha^−1^) led to the highest contribution and CETs had the lowest contribution (42–47.5 g ha^−1^) for enhancing Fe uptake (Table 6). The highest Fe uptake was found in PTR–CDW (5602.8 g ha^−1^) which was statistically at par SRI–SWI; while the values of uptake in ARS–ZTW (5559.8 g ha^−1^) remained inferior to other CETs.

**Table 5:** Influence of crop establishment techniques and nutrient management options on zinc uptake (g ha^−1^) in rice–wheat cropping system during 2013–2014 and 2014–2015.

Treatment	Control	RDN	RDN* + Zn**	75% RDN	75% RDN + Zn	75% RDN + MC1	75% RDN + MC1 + Zn	75% RDN + MC2	75% RDN + MC2 + Zn	Mean
2013–2014										
PTR-CDW	488.3	774.4	856.3	664.0	694.7	754.7	832.1	760.2	844.0	741.0
SRI-SWI	506.5	777.6	840.7	671.0	713.3	757.3	839.9	764.0	841.1	745.7
ARS-ZTW	509.4	800.4	885.7	684.6	712.2	772.1	853.2	781.9	877.0	764.1
Mean	501.4	784.1	860.9	673.2	706.8	761.3	841.7	768.7	854.0	

	Crop establishment techniques	Nutrient management options	Interaction							
SEm±	4.59	6.46	11.19							
CD (*p* = 0.05)	18.02	18.37	31.82							

2014–2015										
PTR–CDW	422.8	725.7	803.3	622.8	645.9	709.3	789.4	714.5	791.0	691.6
SRI–SWI	457.9	722.7	803.9	625.9	660.1	707.0	792.6	711.5	786.3	696.4
ARS–ZTW	465.7	744.1	823.1	639.3	661.8	717.1	797.0	727.1	814.3	709.9
Mean	448.8	730.8	810.1	629.4	655.9	711.1	793.0	717.7	797.2	

	Crop establishment techniques	Nutrient management options	Interaction							
SEm ±	2.66	5.05	8.75							
CD (*p* = 0.05)	10.46	14.36	24.87							

*PTR*, puddled transplanted rice; *SRI*, system of rice intensification; *ARS*, aerobic rice system; *CDW*, conventional drill-sown wheat; *SWI*, system of wheat intensification; *ZTW*, zero tillage wheat; RDN*, recommended dose of nutrients [120 kg nitrogen ha^−1^ and 25.8 kg phosphorus (P) ha^−1^ per crop]; Zn**, 5 kg Zn ha^−1^ through ZnSO_4_·7H_2_O per crop; *MC1*, *Anabaena* sp. (CR1) + *Providencia* sp. (PR3) consortium; *MC2*, *Anabaena*–*Pseudomonas* biofilm formulation; Potassium (K) was applied uniformly in all treatments @ 49.8 kg K ha^−1^ per crop; *Interaction*, significant in both cropping cycle.

**Table 6:** Influence of crop establishment techniques and nutrient management options on iron uptake (g ha^−1^) in rice–wheat cropping system during 2013–2014 and 2014–2015.

Treatment	Control	RDN	RDN* + Zn**	75% RDN	75% RDN + Zn	75% RDN + MC1	75% RDN + MC1 + Zn	75% RDN + MC2	75% RDN + MC2 + Zn	Mean
2013–2014										
PTR-CDW	4598.0	5846.6	6128.2	5368.8	5514.4	5778.0	6015.3	5795.1	6038.6	5675.9
SRI-SWI	4651.1	5869.5	5977.3	5391.1	5547.3	5786.2	6024.7	5805.0	6046.7	5677.7
ARS-ZTW	4492.7	5839.4	6096.2	5312.5	5451.8	5736.0	5971.9	5769.7	6002.0	5630.2
Mean	4580.6	5851.8	6067.2	5357.5	5504.5	5766.7	6004.0	5789.9	6029.1	

	Crop establishment techniques	Nutrient management options	Interaction							
SEm±	6.56	24.02	41.60							
CD (*p* = 0.05)	25.75	68.29	118.29							

2014–2015										
PTR-CDW	4216.8	5708.2	6027.2	5256.2	5347.0	5635.0	5956.2	5669.1	5952.7	5529.8
SRI-SWI	4436.9	5680.1	5998.0	5242.3	5344.6	5600.1	5915.0	5622.6	5944.4	5531.5
ARS-ZTW	4359.3	5676.8	5965.2	5193.1	5303.3	5549.5	5870.2	5603.4	5884.2	5489.5
Mean	4337.7	5688.3	5996.8	5230.5	5331.6	5594.9	5913.8	5631.7	5927.1	

	Crop establishment techniques	Nutrient management options	Interaction							
SEm±	15.17	28.59	49.53							
CD (*p* = 0.05)	59.57	81.30	140.82							

*PTR*, puddled transplanted rice; *SRI*, system of rice intensification; *ARS*, aerobic rice system; *CDW*, conventional drill-sown wheat; *SWI*, system of wheat intensification; *ZTW*, zero tillage wheat; RDN*, recommended dose of nutrients [120 kg nitrogen ha^−1^ and 25.8 kg phosphorus (P) ha^−1^ per crop]; Zn**, 5 kg Zn ha^−1^ through ZnSO_4_·7H_2_O per crop; *MC1*, *Anabaena* sp. (CR1) + *Providencia* sp. (PR3) consortium; *MC2*, *Anabaena*–*Pseudomonas* biofilm formulation; Potassium (K) was applied uniformly in all treatments @ 49.8 kg K ha^−1^ per crop; *Interaction*, significant in both cropping cycle.

### Available soil status of NaHCO_3_-extractable P, 1 N ammonium acetate-extractable K and DTPA-extractable Zn after two cycles of RWCS

The soil P content (NaHCO_3_-extractable) at the end of two cropping cycles of RWCS was higher in RDN, 75% RDN + MC1 and 75% RDN + MC2 with and without Zn application and lower in the treatment 75% RDN with and without Zn and control (Table 7). An increase of 3–9 kg ha^−1^ was recorded, with the highest increase observed with application of 75% RDN + MC1 and the lowest with RDN + Zn. Application of RDN had 6.3–11.3 kg ha^−1^ higher available soil P compared to 75% RDN. Among CETs, both PTR–CDW and SRI–SWI had significantly higher available soil P after two cropping cycles, than ARS–ZTW and the increase in PTR–CDW and SRI–SWI was 1.84–3.12 and 1.75–2.64 kg ha^−1^ compared with ARS–ZTW.

**Table 7:** Influence of crop establishment techniques and nutrient management options on soil phosphorus (kg ha^−1^) (Olsen’s NaHCO_3_-extractable) after completion of first and second cycle of rice–wheat cropping system.

Treatment	Control	RDN	RDN* + Zn**	75% RDN	75% RDN + Zn	75% RDN + MC1	75% RDN + MC1 + Zn	75% RDN + MC2	75% RDN + MC2 + Zn	Mean
2013–2014										
PTR-CDW	11.38	23.57	22.44	17.26	16.80	28.80	27.10	28.46	26.74	22.50
SRI-SWI	10.27	23.76	22.32	17.22	16.87	28.95	26.69	28.32	27.32	22.41
ARS-ZTW	10.58	21.60	19.97	15.54	15.00	26.28	25.48	26.53	24.94	20.66
Mean	10.74	22.98	21.57	16.68	16.22	28.01	26.43	27.77	26.33	

	Crop establishment techniques	Nutrient management options	Interaction							
SEm±	0.13	0.19	0.34							
CD (*p* = 0.05)	0.50	0.55	0.96							

2014–2015										
PTR-CDW	11.78	23.62	21.44	12.38	11.45	27.41	24.28	26.73	23.58	20.30
SRI-SWI	6.68	24.03	21.22	12.32	11.62	27.74	23.49	26.48	24.78	19.82
ARS-ZTW	7.36	20.54	17.35	9.58	8.51	23.61	22.29	24.11	21.24	17.18
Mean	8.61	22.73	20.01	11.43	10.53	26.25	23.35	25.77	23.20	

	Crop establishment techniques	Nutrient management options	Interaction							
SEm±	0.53	0.78	1.35							
CD (*p* = 0.05)	2.07	2.22	3.85							

*PTR*, puddled transplanted rice; *SRI*, system of rice intensification; *ARS*, aerobic rice system; *CDW*, conventional drill-sown wheat; *SWI*, system of wheat intensification; *ZTW*, zero tillage wheat; RDN*, recommended dose of nutrients [120 kg nitrogen ha^−1^ and 25.8 kg phosphorus (P) ha^−1^ per crop]; Zn**, 5 kg Zn ha^−1^ through ZnSO_4_·7H_2_O per crop; *MC1*, *Anabaena* sp. (CR1) + *Providencia* sp. (PR3) consortium; *MC2*, *Anabaena*–*Pseudomonas* biofilm; Potassium (K) was applied uniformly in all treatments @ 49.8 kg K ha^−1^ per crop; *Interaction*, significant in both cropping cycle.

In the present experiment, K was uniformly applied in all the treatments @ 49.8 kg ha^−1^ per crop. Unlike P, available soil K exhibited a consistent reduction in all treatments (Table 8). The rates of N and P application had the highest effect on the soil available K content while Zn fertilization had the lowest effect; however the order of influence was of N and P application > microbial inoculation > CETs > Zn fertilization. The soil available K after first year cropping cycle decreased by 131–147 kg ha^−1^; while after second year, it decreased by 27.6–42.7 kg ha^−1^ over initial available soil K.

**Table 8:** Influence of crop establishment techniques and nutrient management options on soil potassium (kg K ha^−1^) (NH_4_OAC-extractable) after completion of first and second cycle of rice–wheat cropping system.

Treatment	Control	RDN	RDN* + Zn**	75% RDN	75% RDN + Zn	75% RDN + MC1	75% RDN + MC1 + Zn	75% RDN + MC2	75% RDN + MC2 + Zn	Mean
2013–2014										
PTR-CDW	259.9	181.6	169.9	215.0	207.8	188.3	175.9	185.8	172.3	195.2
SRI-SWI	256.5	182.4	174.7	214.5	207.2	189.4	176.1	186.5	173.9	195.7
ARS-ZTW	250.5	165.8	152.9	197.0	190.2	172.1	159.1	169.8	154.5	179.1
Mean	255.6	176.6	165.8	208.8	201.7	183.3	170.4	180.7	166.9	

	Crop establishment techniques	Nutrient management options	Interaction							
SEm±	0.45	2.59	4.49							
CD (*p* = 0.05)	1.76	7.37	12.76							

2014–2015										
PTR-CDW	231.3	146.9	113.1	233.0	217.1	164.2	126.4	156.3	120.2	167.6
SRI-SWI	219.7	151.0	122.2	232.8	217.0	168.5	128.6	159.9	125.1	169.4
ARS-ZTW	205.0	118.0	81.3	197.4	181.6	134.7	95.7	127.3	86.5	136.4
Mean	218.7	138.6	105.5	221.1	205.2	155.8	116.9	147.8	110.6	

	Crop establishment techniques	Nutrient management options	Interaction							
SEm±	1.10	6.06	10.5							
CD (*p* = 0.05)	4.32	17.24	29.87							

*PTR*, puddled transplanted rice; *SRI*, system of rice intensification; *ARS*, aerobic rice system; *CDW*, conventional drill-sown wheat; *SWI*, system of wheat intensification; *ZTW*, zero tillage wheat; RDN*, recommended dose of nutrients [120 kg nitrogen ha^−1^ and 25.8 kg phosphorus (P) ha^−1^ per crop]; Zn**, 5 kg Zn ha^−1^ through ZnSO_4_.7H_2_O per crop; *MC1*, *Anabaena* sp. (CR1) + *Providencia* sp. (PR3) consortium; *MC2*, *Anabaena*–*Pseudomonas* biofilm; Potassium (K) was applied uniformly in all treatments @ 49.8 kg K ha^−1^ per crop; *Interaction*, significant in both cropping cycle.

The soil Zn content (DTPA-extractable) was influenced by Zn fertilization, rate of N and P uptake, microbial inoculation and CETs with highest effect by Zn fertilization and the lowest with microbial inoculation (Table 9). The increase in available soil Zn due to Zn fertilization ranged from 4284.2 to 5361.7 g ha^−1^, with all the three CETs showing an increase in available soil Zn. The variation in Zn content among CETs was 191 and 649.9 g ha^−1^ in first and second year, respectively. The greatest increase in available soil Zn was found with application of 75% RDN + Zn (4950.2 g ha^−1^) applied in PTR–CDW and lowest increase in RDN + Zn (4024.4 g ha^−1^) applied in ARS–ZTW after two cycle of RWCS.

**Table 9:** Influence of crop establishment techniques and nutrient management options on soil DTPA-extractable Zn (g ha^−1^) after completion of first and second cycle of rice–wheat cropping system.

Treatment	Control	RDN	RDN* + Zn**	75% RDN	75% RDN + Zn	75% RDN + MC1	75% RDN + MC1 + Zn	75% RDN + MC2	75% RDN + MC2 + Zn	Mean
2013–2014										
PTR-CDW	2014.7	1809.2	6024.0	1887.1	6142.1	1824.9	6040.8	1820.7	6032.8	3732.9
SRI-SWI	1994.4	1807.7	6035.3	1883.8	6128.7	1823.9	6036.4	1818.7	6035.9	3729.4
ARS-ZTW	1732.5	1524.4	5934.7	1607.1	6063.4	1545.4	5958.4	1538.9	5940.5	3538.4
Mean	1913.8	1713.8	5998.0	1792.7	6111.4	1731.4	6011.9	1726.1	6003.1	

	Crop establishment techniques	Nutrient management options	Interaction							
SEm ±	2.91	4.38	7.59							
CD (*p* = 0.05)	11.44	12.46	21.57							

2014–2015										
PTR-CDW	1659.6	1278.3	6725.8	1388.2	6854.2	1298.7	6736.7	1290.3	6733.3	3773.9
SRI-SWI	1611.4	1281.9	6733.6	1390.1	6841.8	1305.8	6738.9	1296.7	6740.4	3771.2
ARS-ZTW	1104.4	743.0	5928.5	857.3	6064.1	777.6	5949.1	762.1	5929.9	3124.0
Mean	1458.5	1101.0	6462.7	1211.9	6586.7	1127.3	6474.9	1116.4	6467.8	

	Crop establishment techniques	Nutrient management options	Interaction							
SEm ±	3.74	6.86	11.89							
CD (*p* = 0.05)	14.69	19.52	33.81							

*PTR*, puddled transplanted rice; *SRI*, system of rice intensification; *ARS*, aerobic rice system; *CDW*, conventional drill-sown wheat; *SWI*, system of wheat intensification; *ZTW*, zero tillage wheat; RDN*, recommended dose of nutrients [120 kg nitrogen ha^−1^ and 25.8 kg phosphorus (P) ha^−1^ per crop]; Zn**, 5 kg Zn ha^−1^ through ZnSO_4_·7H_2_O per crop; *MC1*, *Anabaena* sp. (CR1) + *Providencia* sp. (PR3) consortium; *MC2*, *Anabaena*–*Pseudomonas* biofilm; Potassium (K) was applied uniformly in all treatments @ 49.8 kg K ha^−1^ per crop; *Interaction*, significant in both cropping cycle.

### Soil chlorophyll and microbial biomass carbon (MBC)

**Figure 1:**
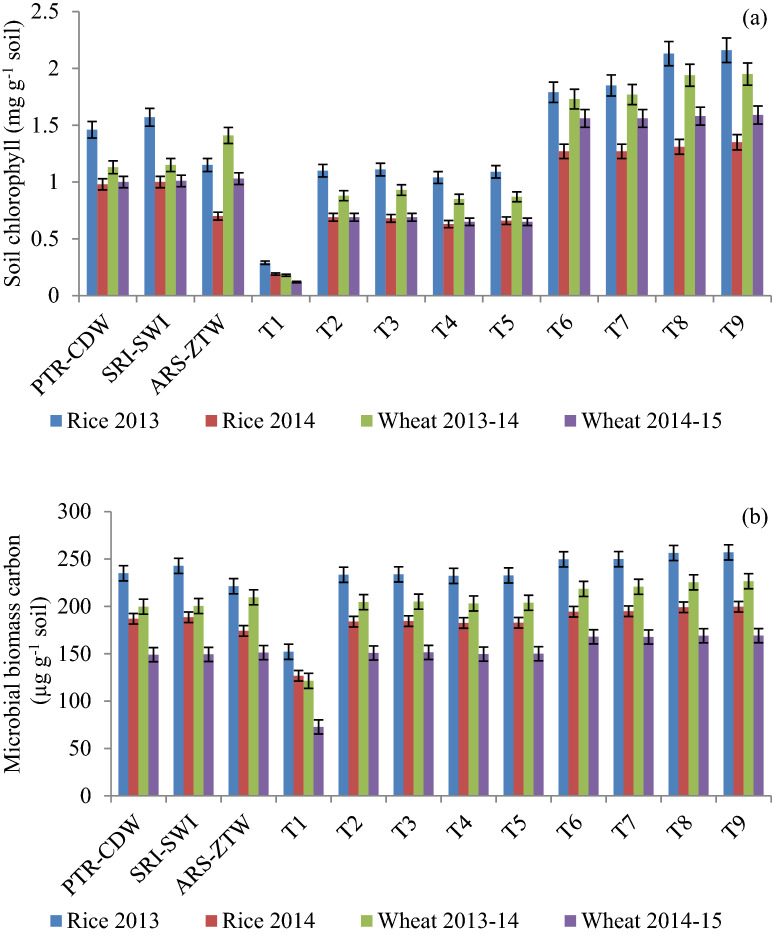
Variation in soil chlorophyll (**a**) and microbial biomass carbon (**b**) in rice and wheat at 60 and 70 days after sowing, respectively due to CETs and nutrient management treatments.

**Figure 2:**
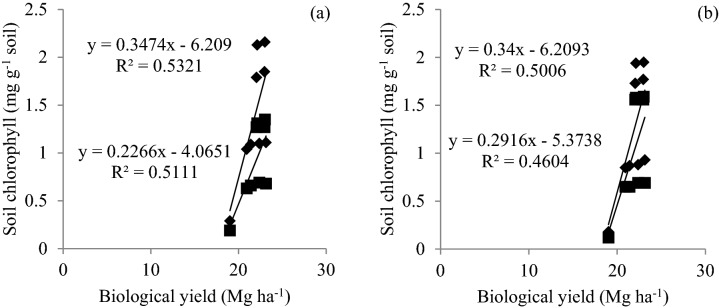
Correlation between biological yield and soil chlorophyll in rice (**a**) at 70 days after sowing in 2013 (◆) and 2014 (■) (DAS) and wheat (**b**) at 60 DAS in 2013–2014 (◆) and 2014–2015 (■).

**Figure 3:**
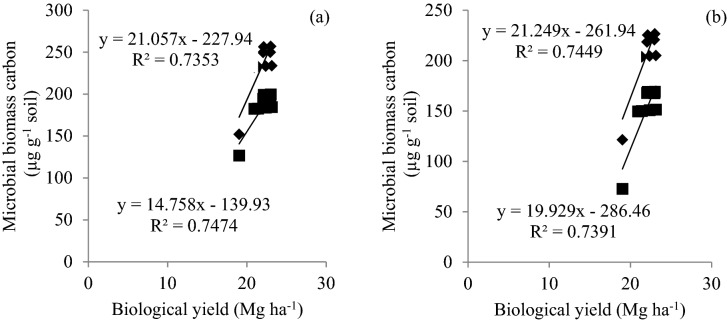
Correlation between biological yield and microbial biomass carbon in rice (**a**) at 70 days after sowing (DAS) in 2013 (◆) and 2014 (■) and wheat (**b**) at 60 DAS in 2013–2014 (◆) and 2014–2015 (■).

All three CETs in rice differed significantly in soil chlorophyll and MBC with significantly higher values of both microbial parameters in SRI during the first year (Fig. 1a,b). During the second year, SRI and PTR remained on par with each other and were significantly superior over ARS. Application of MC2 with 75% RDN led to significantly higher soil chlorophyll and MBC, as compared to the treatment 75% RDN + MC1 in first year and both microbial consortia remained on par in the second year. In wheat, ZTW was found significantly superior in first year and remained on par with other CETs in the second year. Application of 75% RDN + MC2 recorded the highest values of soil chlorophyll and MBC. All the four treatments receiving microbial inoculant had significantly higher soil chlorophyll and MBC than rest of the treatments in both rice and wheat. Both soil chlorophyll and MBC were positively correlated with biological yields (Figs. 2 and 3).

### Partial factor productivity (PFP) and agronomic use efficiency (AUE)

The cropping system PFP for N and P was found significantly higher in ARS–ZTW in both years of study over other CETs (Table 10). The increase in PFP of N in ARS–ZTW was 0.2–0.4 kg grain kg^−1^ N applied; while same for P was 0.8–2.1 kg grain kg^−1^ P applied over other CETs. The PTR–CDW had significantly higher AUE with increase of 1.53–1.55 and 7.1–7.2 kg grain increased kg^−1^ nutrient applied, respectively for N and P over other CETs. Among the nutrient management treatments, PFP was significantly higher in 75% RDN + MC (MC1 or MC2) + Zn than its counterpart without Zn application. All treatments with MC application had significantly higher PFP over RDN and 75% RDN. Application of 75% RDN increased the PFP of N and P by 8.5–9.1 and 39.6–42.3 kg grain kg^−1^ N and P applied over RDN. Zn fertilization increased PFP of N and P by 0.7–2.2 and 3.4–10.3 kg grain kg^−1^ N and P applied; while AUE was 0.73–2.23 and 3.4–10.4 kg grain increased kg^−1^ N and P applied, respectively.

**Table 10:** Effect of crop establishment techniques and nutrient management options on system partial factor productivity and agronomic use efficiency for nitrogen and phosphorus during 2013–2014 and 2014–2015.

Treatment	Nitrogen				Phosphorus			
	Partial factor productivity (kg grain kg^−1^ N applied)		Agronomic use efficiency (kg grain increased kg^−1^ N applied)		Partial factor productivity (kg grain kg^−1^ P applied)		Agronomic use efficiency (kg grain increased kg^−1^ P applied)	
**CETs**	2013–14	2014–15	2013–14	2014–15	2013–14	2014–15	2013–14	2014–15
PTR–CDW	39.9	39.1	8.17	8.65	185.8	181.8	38.0	40.2
SRI–SWI	39.8	38.9	6.62	7.12	184.9	180.9	30.8	33.1
ARS–ZTW	40.2	39.3	6.62	7.12	187.0	182.6	30.8	33.1
SEm ±	0.03	0.03	0.26	0.19	0.16	0.16	1.28	0.78
CD (*P* = 0.05)	0.13	0.13	1.01	0.73	0.62	0.62	5.02	3.06
**Nutrient management options**								
Control	–	–	–	–	–	–	–	–
RDN	36.3	35.2	6.71	6.88	168.6	163.7	31.2	32.0
RDN* + Zn**	37.7	36.8	8.11	8.55	175.2	171.4	37.7	39.7
75% RDN	44.8	44.3	5.37	6.55	208.2	206.0	25.0	30.5
75% RDN + Zn	46.0	45.0	6.60	7.28	213.9	209.4	30.7	33.9
75% RDN + MC1	47.6	46.4	8.18	8.66	221.3	215.8	38.0	40.3
75% RDN + MC1 + Zn	49.8	48.6	10.41	10.89	231.6	226.2	48.4	50.6
75% RDN + MC2	47.8	46.7	8.36	8.94	222.1	217.1	38.9	41.6
75% RDN + MC2 + Zn	49.9	48.7	10.50	10.92	232.1	226.3	48.8	50.8
SEm ±	0.08	0.08	0.12	0.10	0.37	0.37	0.58	0.47
CD (P = 0.05)	0.23	0.23	0.36	0.29	1.05	1.05	1.66	1.34
Interaction	Sig	NS	Sig	Sig	Sig	Sig	Sig	Sig

*PTR*, Puddled transplanted rice; *SRI*, System of rice intensification; *ARS*, Aerobic rice system; *CDW*, Conventional drill-sown wheat; *SWI*, System of wheat intensification; *ZTW*, Zero tillage wheat; RDN*, Recommended dose of nutrients [120 kg nitrogen ha^–1^ and 25.8 kg phosphorus (P) ha^–1^ per crop]; Zn**, 5 kg Zn ha^–1^ through ZnSO_4_.7H_2_O per crop; *MC1*, *Anabaena* sp. (CR1) + *Providencia* sp. (PR3) consortium; *MC2*, *Anabaena*–*Pseudomonas* biofilm; Potassium (K) was applied uniformly in all treatments @ 49.8 kg K ha^–1^ per crop; *Sig*, Significant; *NS*, Non-significant.

### Cropping system nutrient balance

Out of the total available P present in soil (soil initial available P + P applied through fertilizer) 36.9–40.8% was accounted by plant uptake and 7.6–8.7% contributed to NaHCO_3_-extractable soil P; while 50.4–55.3% was not extracted by NaHCO_3_ (Table 11). This 50.4–55.3% P (not extracted by NaHCO_3_) may be present in soil in fixed form or part of it might have been lost from the soil, due to leaching. The fixed P needs to be reutilized to make P fertilization in RWCS economical and application of microbial consortia is a suitable option. The difference between calculated and actual balance was 13.2–35.5 kg ha^−1^ and 24.3–32.0 kg ha^−1^, respectively in the first and second year in treatments with application of RDN and higher values recorded in calculated balance in both years.

**Table 11:** Effect of crop establishment techniques and nutrient management options on phosphorus (P) (NaHCO_3_-extractable) (kg ha^−1^) content in soil in RWCS.

Treatment	Initial NaHCO_3_-extractable P		P applied through fertilizer		Total initial P present in soil		Cropping system P uptake		Balance after completion of cropping cycle		Actual P present in soil after completion of cropping cycle	
	2013–14	2014–15	2013–14	2014–15	2013–14	2014–15	2013–14	2014–15	2013–14	2014–15	2013–14	2014–15
**Crop establishment techniques**												
PTR–CDW	17.0	22.5	51.6	51.6	68.6	74.1	26.9	26.6	41.7	47.5	22.5	20.3
SRI–SWI	17.0	22.4	51.6	51.6	68.6	74.0	26.9	26.7	41.7	47.3	22.4	19.8
ARS–ZTW	17.0	20.7	51.6	51.6	68.6	72.3	27.8	27.4	40.8	44.9	20.7	17.2
SEm ±	–	0.13	–	–	–	0.13	0.10	0.11	0.10	0.23	0.13	0.53
CD (*P* = 0.05)	–	0.50	–	–	–	0.50	0.38	0.41	0.38	0.91	0.50	2.07
**Nutrient management options**												
Control	17.0	10.7	51.6	51.6	68.6	62.3	22.4	21.7	46.2	40.6	10.7	8.6
RDN	17.0	23.0	51.6	51.6	68.6	74.6	28.0	27.6	40.6	47.0	23.0	22.7
RDN* + Zn**	17.0	21.6	51.6	51.6	68.6	73.2	29.1	28.9	39.5	44.3	21.6	20.0
75% RDN	17.0	16.7	51.6	51.6	68.6	68.3	25.8	25.8	42.8	42.5	16.7	11.4
75% RDN + Zn	17.0	16.2	51.6	51.6	68.6	67.8	26.4	26.2	42.2	41.6	16.2	10.5
75% RDN + MC1	17.0	28.0	51.6	51.6	68.6	79.6	27.4	27.2	41.2	52.4	28.0	26.3
75% RDN + MC1 + Zn	17.0	26.4	51.6	51.6	68.6	78.0	28.8	28.5	39.8	49.5	26.4	23.4
75% RDN + MC2	17.0	27.8	51.6	51.6	68.6	79.4	27.6	27.5	41.0	51.9	27.8	25.8
75% RDN + MC2 + Zn	17.0	26.3	51.6	51.6	68.6	77.9	28.9	28.6	39.7	49.3	26.3	23.2
SEm ±	–	0.19	–	–	–	0.19	0.15	0.16	0.15	0.36	0.19	0.78
CD (P = 0.05)	–	0.55	–	–	–	0.55	0.42	0.47	0.42	1.02	0.55	2.22
Interaction	–	Sig	–	–	–	Sig	Sig	Sig	Sig	Sig	Sig	Sig

*PTR*, Puddled transplanted rice; *SRI*, System of rice intensification; *ARS*, Aerobic rice system; *CDW*, Conventional drill-sown wheat; *SWI*, System of wheat intensification; *ZTW*, Zero tillage wheat; RDN*, Recommended dose of nutrients [120 kg nitrogen ha^–1^ and 25.8 kg phosphorus (P) ha^–1^ per crop); Zn**, 5 kg Zn ha^–1^ through ZnSO_4_.7H_2_O per crop; *MC1*, *Anabaena* sp. (CR1) + *Providencia* sp. (PR3) consortium; *MC2*, *Anabaena*–*Pseudomonas* biofilm; *Sig*, Significant; *NS*, Non-significant; Potassium (K) was applied uniformly in all treatments @ 49.8 kg K ha^–1^ per crop.

In the case of potassium, both calculated and actual balance was negative over initial soil available K in both years (Table 12). The total cropping system K uptake across different CETs in first year varied between 223 and 230.8 kg ha^−1^, which was higher than K applied in RWCS with calculated and actual negative balance of 124–131 kg ha^−1^ and 131–148 kg ha^−1^, over initial soil available K respectively. During the second year, total cropping system K uptake, calculated negative balance and actual negative balance were 216–225, 116.13 and 26–42 kg ha^−1^, respectively. Out of the total available Zn present in soil (soil initial available Zn + Zn applied through fertilizer) across different CETs, only 11.6–18.5% were taken up by plants (Table 13); while 54.2–90.5% contributed to increase in soil DTPA-extractable Zn content of soil. The actual available Zn balance after completion of two cropping cycles of RWCS was lower by 1484–1927 g ha^−1^ than calculated balance.

**Table 12:** Effect of crop establishment techniques and nutrient management options on potassium (K) (NH_4_OAC-extractable) (kg K ha^–1^) content in soil in RWCS.

Treatment	Initial NH_4_OAC-extractable K		K applied through fertilizer		Total initial K present in soil		Cropping system K uptake		Balance after completion of cropping cycle		Actual K present in soil after completion of cropping cycle	
	2013–14	2014–15	2013–14	2014–15	2013–14	2014–15	2013–14	2014–15	2013–14	2014–15	2013–14	2014–15
**Crop establishment techniques**												
PTR–CDW	327.0	195.2	99.6	99.6	426.6	294.8	223.8	216.7	202.8	78.0	195.2	167.6
SRI–SWI	327.0	195.7	99.6	99.6	426.6	295.3	223.3	215.9	203.3	79.4	195.7	169.4
ARS–ZTW	327.0	179.1	99.6	99.6	426.6	278.7	230.8	225.7	195.8	53.0	179.1	136.4
SEm ±	–	0.45	–	–	–	0.45	0.46	0.65	0.46	1.06	0.45	1.10
CD (*P* = 0.05)	–	1.76	–	–	–	1.76	1.80	2.55	1.80	4.15	1.76	4.32
**Nutrient management options**												
Control	327.0	255.6	99.6	99.6	426.6	355.2	156.3	149.2	270.3	206.0	255.6	218.7
RDN	327.0	176.6	99.6	99.6	426.6	276.2	240.0	231.3	186.6	44.9	176.6	138.6
RDN* + Zn**	327.0	165.8	99.6	99.6	426.6	265.4	251.1	245.3	175.5	20.2	165.8	105.5
75% RDN	327.0	208.8	99.6	99.6	426.6	308.4	206.7	203.5	219.9	105.0	208.8	221.1
75% RDN + Zn	327.0	201.7	99.6	99.6	426.6	301.3	214.1	207.5	212.5	93.9	201.7	205.2
75% RDN + MC1	327.0	183.3	99.6	99.6	426.6	282.9	233.2	225.4	193.4	57.5	183.3	155.8
75% RDN + MC1 + Zn	327.0	170.4	99.6	99.6	426.6	270.0	246.4	241.6	180.2	28.4	170.4	116.9
75% RDN + MC2	327.0	180.7	99.6	99.6	426.6	280.3	235.8	229.0	190.8	51.4	180.7	147.8
75% RDN + MC2 + Zn	327.0	166.9	99.6	99.6	426.6	266.5	250.1	242.3	176.5	24.2	166.9	110.6
SEm ±	–	2.59	–	–	–	2.59	2.61	2.59	2.61	5.13	2.59	6.06
CD (P = 0.05)	–	7.37	–	–	–	7.37	7.43	7.38	7.43	14.58	7.37	17.24
Interaction	–	Sig	–	–	–	Sig	NS	Sig	NS	NS	Sig	Sig

*PTR*, Puddled transplanted rice; *SRI*, System of rice intensification; *ARS,* Aerobic rice system; *CDW*, Conventional drill-sown wheat; *SWI*, System of wheat intensification; *ZTW*, Zero tillage wheat; RDN*, Recommended dose of nutrients [120 kg nitrogen ha^–1^ and 25.8 kg phosphorus (P) ha^–1^ per crop]; Zn**, 5 kg Zn ha^–1^ through ZnSO_4_.7H_2_O per crop; *MC1*, *Anabaena* sp. (CR1) + *Providencia* sp. (PR3) consortium; *MC2*, *Anabaena*–*Pseudomonas* biofilm; Potassium (K) was applied uniformly in all treatments @ 49.8 kg K ha^–1^ per crop; *Sig*, Significant; *NS*, Non-significant.

**Table 13:** Effect of crop establishment techniques and nutrient management options on zinc (Zn) (DTPA-extractable) (g ha^–1^) content in soil in RWCS.

Treatment	Initial DTPA-extractable Zn		Zn applied through fertilizer		Total initial available Zn present in soil		Cropping system Zn uptake		Balance after completion of cropping cycle		Actual available Zn present in soil after completion of cropping cycle	
	2013–14	2014–15	2013–14	2014–15	2013–14	2014–15	2013–14	2014–15	2013–14	2014–15	2013–14	2014–15
**Crop establishment techniques**												
PTR–CDW	1904.0	3732.9	2222.2	2222.2	4126.2	5955.1	741.0	691.6	3385.3	5263.5	3732.9	3773.9
SRI–SWI	1904.0	3729.4	2222.2	2222.2	4126.2	5951.6	745.7	696.4	3380.5	5255.2	3729.4	3771.2
ARS–ZTW	1904.0	3538.4	2222.2	2222.2	4126.2	5760.6	764.1	709.9	3362.2	5050.6	3538.4	3124.0
SEm ±	-	2.91	-	-	-	2.91	4.59	2.66	4.59	5.53	2.91	3.74
CD (*P* = 0.05)	-	11.44	-	-	-	11.44	18.02	10.46	18.02	21.70	11.44	14.69
**Nutrient management options**												
Control	1904.0	1913.8	0.0	0.0	1904.0	1913.8	501.4	448.8	1402.6	1465.0	1913.8	1458.5
RDN	1904.0	1713.8	0.0	0.0	1904.0	1713.8	784.1	730.8	1119.9	982.9	1713.8	1101.0
RDN* + Zn**	1904.0	5998.0	5000.0	5000.0	6904.0	10,998.0	860.9	810.1	6043.1	10,187.9	5998.0	6462.7
75% RDN	1904.0	1792.7	0.0	0.0	1904.0	1792.7	673.2	629.4	1230.8	1163.3	1792.7	1211.9
75% RDN + Zn	1904.0	6111.4	5000.0	5000.0	6904.0	11,111.4	706.8	655.9	6197.2	10,455.5	6111.4	6586.7
75% RDN + MC1	1904.0	1731.4	0.0	0.0	1904.0	1731.4	761.3	711.1	1142.7	1020.3	1731.4	1127.3
75% RDN + MC1 + Zn	1904.0	6011.9	5000.0	5000.0	6904.0	11,011.9	841.7	793.0	6062.3	10,218.9	6011.9	6474.9
75% RDN + MC2	1904.0	1726.1	0.0	0.0	1904.0	1726.1	768.7	717.7	1135.3	1008.4	1726.1	1116.4
75% RDN + MC2 + Zn	1904.0	6003.1	5000.0	5000.0	6904.0	11,003.1	854.0	797.2	6050.0	10,205.9	6003.1	6467.8
SEm ±	-	4.38	-	-	-	4.38	6.46	5.05	6.46	9.07	4.38	6.86
CD (P = 0.05)	-	12.46	-	-	-	12.46	18.37	14.36	18.37	25.79	12.46	19.52
Interaction	-	Sig	-	-	-	Sig	Sig	Sig	Sig	Sig	Sig	Sig

*PTR*, Puddled transplanted rice; *SRI*, System of rice intensification; *ARS*, Aerobic rice system; *CDW*, Conventional drill-sown wheat; *SWI*, System of wheat intensification; *ZTW*, Zero tillage wheat; RDN*, Recommended dose of nutrients [120 kg nitrogen ha^–1^ and 25.8 kg phosphorus (P) ha^–1^ per crop]; Zn**, 5 kg Zn ha^–1^ through ZnSO_4_.7H_2_O per crop; *MC1*, *Anabaena* sp. (CR1) + *Providencia* sp. (PR3) consortium; *MC2*, *Anabaena*–*Pseudomonas* biofilm; Potassium (K) was applied uniformly in all treatments @ 49.8 kg K ha^–1^ per crop; *Sig*, Significant.

## Discussion

### Biological yield of cropping system

The variation in biological yield at individual crop level was negligible which led to also the same at cropping system level. In case of rice, weed infestation problem and also the problem of seedling establishment due to high temperature and lower number of fertile tillers in ARS made it significantly inferior over PTR and SRI; while residual effect (especially of nutrients) of previous season ARS, better plant stand establishment and higher values of yield attributes made ZTW superior over other CETs of wheat. Among nutrient management options, the inherent soil nutrient status, nutrient application rate, yield enhancement due to microbial consortia and plant nutrient acquisition capacity influences the significance of applied treatments. This is clear from yield enhancement recorded in terms of cropping system in treatment with application of RDN by 1.43–1.24 Mg ha^−1^ and 3.35–3.62 Mg ha^−1^ over 75% RDN and control, respectively. The positive effect of applying RDN and Zn fertilization on crop yield was also earlier reported by Shivay et al.^40^ and Singh et al.^43^. The contributions of both types of microbial inoculation to cropping system yield was highest in ARS–ZTW (1.14 and 1.19 Mg ha^−1^) than the other CET systems in the first year; while during the second year, both microbial inoculants performed better in PTR–CDW with increase in cropping system yield by1.03 and 1.16 Mg ha^−1^, respectively due to application of MC1 and MC2. One of the reasons for this difference was variation in rainfall and other weather parameters across years. The total rainfall received during rice growing season in first year was 1349 mm; while during the second year, it was 451.4 mm. This higher rainfall leads to higher water level in rice field, which may have favoured and positively influenced the survival and nutrient release/acquisition capacity of the applied inoculants. This is validated from the higher values of microbial biomass carbon and soil chlorophyll during the first year (Fig. 1a,b).

### Cropping system N, P and K uptake

The cropping system N uptake in 75% RDN + MC1 + Zn and 75% RDN + MC2 + Zn was on par with application of 100% RDN, illustrating the significant role of microbial inoculation in nitrogen nutrition in RWCS. The superiority of RDN over 75% RDN signifies the role of optimal and balanced fertilization. The increased level of biological nitrogen fixation due to presence of optimum population of *Anabaena* sp., low available soil N and suboptimal N addition (75% of recommended) through fertilization are the important reasons for increasing N uptake in inoculated treatments. The nitrogen fixation in cyanobacteria (*Anabaena* sp.) takes place in specialized heterocyst cells. These cells create microanaerobic environment to form proper functioning of nitrogenase enzyme (enzyme involved in biological nitrogen fixation). The significance of microbial inoculation in increasing to nitrogen uptake in rice^44^ and wheat^45^ as well as contribution of microbial inoculation to growth and yielding ability of rice^46^ and wheat^47^ was reported. In present study, increase in N uptake in ARS–ZTW was same as that of PTR–CDW and SRI–SWI showing that, MC1 and MC2 also found better for application in ARS–ZTW (aerobic condition).

The phosphorus is second most important nutrient after N; while need and significance of potassium nutrition in RWCS was also reported^48^. The conversion of applied water soluble P from fertilizer to the form which was not available to the plant and its fixation in insoluble form are the area where applied microbial inoculation work. The production of organic acids and lowering soil pH due to organic acid as the mechanisms by which soil fixed P was made available for plant growth by microbial cultures. The role of microbes in P solubilisation and mobilization was reported by Alori et al.^49^. This ultimately leads to improvement in crop growth, yielding ability and P uptake^50^ as observed in our study. The P uptake was found higher in 75% RDN + MC1 or MC2 over 75% RDN even though rate of P application was remained same in these treatments. This was due to better nutrient acquisition capacity of well grown plant than nutrient stressed plant. In case of K, adoption of ZTW may increase the opportunity for incorporation and retention of straw^3^ thereby reduces the problem of delay in sowing and burning of residue.

### Cropping system Zn and Fe uptake

Both Zn and Fe uptake of rice and wheat are important considering their role in plant and human/animal health^51^. In present investigation, nutrient application rate of N and P had the highest contribution to increase in Zn uptake. This was due to their higher contribution to biological yield than rest factors. The soil Zn application rate was 5 kg ha^−1^ which is very high than cropping system Zn uptake; hence there will be sufficient Zn available for the plant uptake. In such conditions, the uptake capacity of plant (dry matter production) will decide the Zn uptake. The application of primary nutrient such as N and P has higher contribution to dry matter production; hence uptake of Zn in our study was mainly decided N and P application rate; even though concentration of Zn in rice and wheat in both years of study was found influenced mainly by Zn fertilization (data of concentration was not shown). This showed that dry matter production played major role in deciding Zn uptake than concentrations in present study. Another factors governing variation in Zn and Fe uptake is CETs. The variation in Zn uptake across studied CETs is due to change in hydrological regime^52,53^, variation in growth vigour and yield^15,27,54^, soil inherent nutrient availability and variation in conversion of applied Zn in different forms.

### Soil available P (NaHCO_3_-extractable), K (NH_4_OAC-extractable) and Zn (DTPA-extractable) status

The variation in soil NaHCO_3_-extractable P status in present study was contributed by higher uptake in ARS–ZTW, increase in soil available P (occluded P) under puddled condition in PTR and SRI, contribution of microbial consortia, rate of P application (100% and 75% RDN) and less vigorous growth of wheat in CDW and SWI leading lower P acquisition. The variation in available soil P status across CETs was also reported by Pradhan et al.^55^. The increase in soil available P with application of microbial inoculation^56^ was significantly higher than RDN application even though uptake was remained on par. This showed that, microbial inoculations is sustainable strategy and have capacity to increase the contribution of fertilizer applied P and soil inherent but unavailable P to plant P uptake. At the same time, only 75% of RDN was applied with microbial inoculation and leads to higher nutrient use efficiency of P fertilizer^57^ as yield level was same as that observed with RDN.

In rice and wheat 89–91% and 85–89% of K remained is straw; therefore, its recycling is possible either through residue retention or residue incorporation. The lower response of rice and wheat to K application^58^ due to higher NH_4_OAC-extractable K content in trans and upper Indo-Gangetic plain^48^ and higher subsidy on N and P than K are the possible reasons for lower K application in RWCS. This ultimately leads to imbalanced fertilization. This imbalance can be seen from nutrient application ratio (4.9:2.2:1; N:P_2_O_5_:K_2_O in rice and 11.7:4.9:1; N:P_2_O_5_:K_2_O in wheat)^30^. In such condition, zero tillage wheat with residue incorporation and/or retention will be best option which helps in nutrient cycling and also ensuring timely sowing.

The application of 10 kg Zn ha^−1^ through ZnSO_4_·7H_2_O in single cropping cycle of RWCS increases the soil DTPA-extractable Zn along with increasing Zn uptake^59^. This increased DTPA-extractable Zn going to pay to next crops in succession. One of the possible reasons for increasing soil Zn content was the difference between Zn uptake in RWCS and amount applied. In present study, the uptake of Zn varied between 691 and 764 g ha^−1^ (6–9% of the total Zn applied in one cropping cycle of RWCS) in single cycle of RWCS. This showed that, 9200–9300 g Zn ha^−1^ remained unutilized; while increase in soil DTPA-extractable Zn content was 3100–3700 g ha^−1^ (42–54% of the total Zn applied in one cycle of RWCS). The remaining quantities either get fixed in the form which is not extracted by DTPA or form chemical compounds with other elements observed in soil^60^.

### Soil microbial properties

The soil microbial properties respond quickly and significantly to change in tillage^61^, crop establishment techniques^29,62^, fertilization^63^ and external inoculation of microbial culture^64^. In the present study, microbial consortia involving cyanobacteria for nitrogen fixation and bacteria for P solubilization were used. Their growth and activity in soil, illustrates their promise in competing with the inherent soil microbial population and responding to CETs and fertilization. The use of microbial consortia or microbial biofilms has immense significance as the synergy among the partners helps in efficient proliferation and functioning under different temperature-light regimes or environmental fluctuations, including flooding or dry conditions as they can grow attached to soil particles or plant roots or flood water^65–67^. Cyanobacteria are thoroughly investigated for their role as nitrogen-fixers, plant growth promoters and their benefits to neighbouring microflora and fauna, thereby, contributing to improved plant health and soil fertility. Bacteria such as *Providencia*, or *Pseudomonas* employ a variety of solubilization reactions, such as acidification, chelation, exchange reactions, and production of gluconic acid, to release soluble from insoluble P. Cyanobacterium-based combinations as consortia with *Providencia,* known for its nutrient-enriching potential in rice wheat cropping system^65,67,68^, and as biofilm with *Pseudomonas* sp. which has shown promise for its P mobilising and plant-promoting traits, is also well documented^66,67^. The superiority of SRI and PTR over ARS was might be due to better growth condition (puddled soil and continuous saturated condition of soil) for the applied microbial consortia than that of ARS; while in wheat, higher organic matter from stubbles of previous season rice crop and better soil physical conditions (no soil puddling in ARS) can be the important reasons for higher values of soil chlorophyll and MBC in ZTW. Significant improvement in the biological properties illustrates the potential of the applied microbes in mobilising nutrients and enhancing their uptake by plants.

### Partial factor productivity (PFP) and agronomic use efficiency (AUE) of N and P

The higher PFP in ARS–ZTW was due to higher cropping system yield with the same level of N and P applied. In case of AUE, the superiority of PTR–CDW can be due to lower yield in control plot, than in control plot of SRI–SWI and ARS–ZTW. This indicates higher availability of soil nutrients and overall contribution in SRI–SWI and ARS–ZTW systems. Among nutrient management options, AUE and PFP of N and P were significantly affected by the rate of nutrient application, microbial consortia and Zn fertilization. The significantly higher PFP and AUE with application of microbial consortia was due to lower rate of application over RDN, higher yield over 75% RDN and contribution of biological nitrogen fixation and P solubilisation to N and P uptake. The contribution of microbial consortia to increase in PFP of N and P was 2.1–3.0 and 9.8–13.9 kg grain kg^−1^ nutrient applied and same for AUE was 2.1–2.9 and 9.8–13.9 kg grain increased kg^−1^ nutrient applied. This improvement in PFP and AUE by microbial consortia without yield penalty is highly important as rice and wheat together consume 52.4% N and 48.4% of P out of total consumption in India^30^.

### Nutrient balance

Analyses of the balance of P, K and Zn showed that the application of recommended rate of P and Zn had a positive effect on their available soil status; while application of K at recommended rate is not sufficient for RWCS. The order of significance of applied treatments in increasing soil P after completion of two cropping cycle of RWCS over initial available soil P was—application of microbial consortia > rate of N and P application > CETs with respective contribution of 8.8–9.3, 5.7 and 0.2–3.3 kg ha^−1^, respectively. This order of significance showed the important contributions of microbial consortia and their application towards efficient P nutrition of RWCS. The order of significance of applied treatments on K uptake and soil available K status was contradictory. The results showed that the variation in soil available K status was mainly governed by plant nutrient uptake. The application of Zn, with 75% RDN showed the highest increase in soil available Zn; while uptake was highest with RDN + Zn. The actual Zn balance after completion of two cropping cycles of RWCS was lower than calculated balance signifying the possible conversion of applied Zn into forms, not extracted by DTPA i.e. unavailable pool of Zn.

Our study showed that the uptake of nitrogen, phosphorus, potassium, zinc and iron in terms of cropping system was significantly influenced by CETs, microbial inoculation, zinc fertilization and rate of N and P fertilization in both the years of study. Among these factors, the rate of N and P application brought about the maximum effect, while effect of CETs was minimal for all nutrients. In case of nitrogen and potassium uptake, the order of significance was rate of N and P application > Zn fertilization > microbial inoculation > CETs; while for phosphorus and zinc uptake, rate of N and P application > microbial inoculation > Zn fertilization > CETs. The positive effect of Zn fertilization on soil DTPA-extractable Zn and nitrogen uptake along with increasing cropping system yield and Zn uptake was also distinct in the present study. The superior performance of the microbial consortia used in the present study in terms of cropping system yield as well as for N and P uptake in ARS–ZTW, highlights their promise in actively participating and helping in nutrient acquisition under aerobic conditions.

## Methods

### Experimental site

A study was conducted at Research Farm of ICAR–Indian Agricultural Research Institute, New Delhi, India (28° 38′ N, 77° 10′ E and 228.6 m above mean sea level) (Arabian Sea). The climate of Delhi is of sub-tropical and semi-arid type with hot and dry summer and cold winter and falls under the agro-climatic zone ‘Trans-Indo-Gangetic plains’. The mean annual normal rainfall and pan evaporation is 650 mm and 850 mm, respectively. Total amount of rainfall received during the growing duration of first (2013–2014) and second (2014–2015) cropping cycle of RWCS was 1497.4 mm and 760 mm. In first cropping cycle, 1349.8 mm was received during rice growing season and 147.6 mm was received during wheat growing season; while the same for second cropping cycle was 451.4 and 308.6 mm, respectively. The soil of experimental field (15 cm soil depth) was sandy clay loam in texture having pH 7.6 and organic carbon of 5.4 g kg^−1^ soil. The amount of alkaline KMnO_4_-extractable N, NaHCO_3_-extractable P, 1 N ammonium acetate-extractable K and DTPA-extractable Zn was 257 kg ha^−1^, 17 kg ha^−1^, 327 kg ha^−1^ and 0.85 mg kg^−1^ soil, respectively.

### Experimental details

The rice variety ‘Pusa Sugandh 5′ and wheat variety ‘HD 2967’ were planted in experiment which was conducted in split-plot design with treatment details as mentioned in Table 14 and all the treatments were replicated thrice.

**Table 14:** Treatment details applied in rice–wheat cropping system and respective abbreviations used throughout the text.

Sl. No.	Treatments	Short form used in tables and text
**Main plot treatments (net plot area 76.14 m**^**2**^**)**		
1	Puddled transplanted rice followed by conventional drill-sown wheat	PTR-CDW
2	System of rice intensification followed by system of wheat intensification	SRI-SWI
3	Aerobic rice system followed by zero tillage wheat	ARS-ZTW
**Sub-plot treatment (net plot area 8.46 m**^**2**^**)**		
1	Absolute control (no fertilizer application)	Control (T1)
2	100% recommended dose of nutrients* (nitrogen and phosphorus)	RDN (T2)
3	100% recommended dose of nutrients (nitrogen and phosphorus) + Zinc**	RDN + Zn (T3)
4	75% recommended dose of nutrients (nitrogen and phosphorus)	75% RDN (T4)
5	75% recommended dose of nutrients (nitrogen and phosphorus) + Zinc	75% RDN + Zn (T5)
6	75% recommended dose of nutrients (nitrogen and phosphorus) + *Anabaena* sp. (CR1) + *Providencia* sp. (PR3) consortium (MC1)	75% RDN + MC1 (T6)
7	75% recommended dose of nutrients (nitrogen and phosphorus) + *Anabaena* sp. (CR1) + *Providencia* sp. (PR3) consortium (MC1) + Zinc	75% RDN + MC1 + Zn (T7)
8	75% recommended dose of nutrients (nitrogen and phosphorus) + *Anabaena*–*Pseudomonas* biofilm (MC2)	75% RDN + MC2 (T8)
9	75% recommended dose of nutrients (nitrogen and phosphorus) + *Anabaena*–*Pseudomonas* biofilm (MC2) + Zinc	75% RDN + MC2 + Zn (T9)

100% recommended dose of nutrients*: 120 kg nitrogen ha^–1^ and 25.8 kg phosphorus (P) ha^–1^ per crop; Zn**: 5 kg Zn ha^–1^ through ZnSO_4_.7H_2_O per crop; Potassium (K) was applied uniformly in all treatments @ 49.8 kg K ha^–1^ per crop; Chemical fertilizer used for N, P and K were urea, single super phosphate and muriate of potash, respectively. Both MC1 and MC2 were applied as formulations prepared using paddy straw compost: vermiculite as a carrier.

### Crop management in different CETs

In order to have same crop growth duration in all CETs, sowing of rice in main field for ARS and sowing of rice seeds in nursery for transplanting in both PTR and SRI were done on same date (16th and 17th June in first year and 19th and 20th June in second year). Similarly, sowing of wheat in all three CETs were also done on same date (15th and 16th November in first year and 17th and 18th November in second year). The details of crop management in different CETs are given in Table 15. The details of the preparation of microbial inoculants and their formulations are given by Prasanna et al.^65^, Nain et al.^66^ and Prasanna et al.^67^. Both these formulations used in study (Table 14) were prepared by mixing with vermiculite (hydrous phyllosilicate mineral): compost (1:1) as the carrier. The paddy straw compost has C/N ratio of 16.22:1 and humus content of 13.8% (pH 7.34). The cyanobacterial and bacterial colony forming units in the formulations was 10^4^ and 10^8^ g^−1^ carrier, respectively, as optimized in earlier studies^68^.

**Table 15:** Details of crop management in each crop establishment technique in rice and wheat.

Sl. no.	Particular	Crop establishment techniques (CETs)		
	Rice	PTR	SRI	ARS
1	Field preparation	One ploughing, 2 harrowing followed by puddling after application of 10 and 12 cm depth of water, respectively in first and second year	One ploughing, 2 harrowing followed by puddling after application of 10 cm depth of water in both year	One ploughing, 2 harrowing and planking after pre-sowing irrigation of 5 cm
2	Seed and sowing	Seed rate: 20 kg ha^−1^ Spacing: 20 cm × 15 cm Sowing method: transplanting of 2–3 seedling at each hill (23–25 days old)	Seed rate: 5 kg ha^−1^ Spacing: 20 cm × 20 cm Sowing method: transplanting of 1 seedling at each hill (13–14 days old)	Seed rate: 60 kg ha^−1^ Spacing: 20 cm (row to row) Sowing method: drilling (direct sowing)
3	Water management	Application of irrigation with 5 cm depth as and when water disappeared from the surface at each irrigation	Saturated field condition was maintained; irrigation applied when fine cracks were developed; Depth of water application at each irrigation: 3 cm up to flowering and 5 cm from flowering to grain filling	Aerobic condition throughout the crop growth; Available soil moisture depletion (ASMD) approach for irrigation; irrigation at 50% ASMD; Depth of irrigation: 3 cm up to flowering and 5 cm from flowering to grain filling
4	Weed management	Two hand weeding at 20 and 40 days after transplanting (DAT)	Two hand weeding at 20 and 40 days after transplanting	Three hand weeding at 15, 30 and 45 days after sowing (DAS)
5	Nutrient management	Rate of application: as per the treatment details mentioned in Table 14 Methods and timing of application: Incorporation of P, K and Zn just before transplanting and broadcasting of N in three equal splits at 5, 25 and 45 DAT	Rate of application: as per the treatment details mentioned in Table 14 Methods and timing of application: Incorporation of P, K and Zn just before transplanting and broadcasting of N in three equal split at 5, 25 and 45 DAT	Rate of application: as per the treatment details mentioned in Table 14 Methods and timing of application: drilling below the seed for 1/3^rd^ N and whole quantity of P, K and Zn at the time of sowing and broadcasting for top dressing of nitrogen1/3^rd^ N each at 30 and 60 DAS
6	Application of microbial inoculation	Slurry of microbial cultures was made by using water along with 1% Carboxymethyl cellulose (CMC) as a sticker and seedlings were dipped in this slurry for 30 min before transplanting	Slurry of microbial cultures was made by using water along with 1% CMC as a sticker and seedlings were dipped in this slurry for 30 min before transplanting	The pre-soaked seeds were treated with thick slurry of microbial cultures, using 1% CMC as a sticker for 30 min and seeds allowed to dry in shade for 30 min before sowing

	Wheat	CDW	SWI	ZTW
1	Field preparation	One ploughing by following disc harrow, another ploughing with cultivator and planking	One ploughing by following disc harrow, another ploughing with cultivator and planking	No tillage operation except reshaping of bunds and direct sowing was done
2	Seed and sowing	Seed rate: 100 kg ha^−1^ Spacing: 22.5 cm (row to row) Sowing method: Drilling	Seed rate: 30 kg ha^−1^ Spacing: 20 cm × 20 cm Sowing method: Dibbling (1–2 seeds at each spot)	Seed rate: 120 kg ha^−1^ Spacing: 20 cm (row to row) Sowing method: Drilling
3	Water management	Critical crop growth stages approach method was adopted in all CETs; Irrigation was given at six critical crop growth stages viz., crown root initiation, tillering, late jointing, flowering, milking and grain hardening stages		
4	Weed management	Two hand weeding at 20–25 and 40–45 DAS was done in all CETs		
5	Nutrient management	Rate of application: as per the treatment details mentioned in Table 14 Methods and timing of application: Drilling of 1/3rd N, complete dose of P, K and Zn below the seed at the time of sowing; top dressing of 1/3rd N each at 30 and 60 DAS in all CETs		
6	Application of microbial inoculations	The seeds were treated with slurry of respective microbial inoculant prepared using water and 1% CMC for 30 min and seeds were shade dried for 30 min		

### Observations recorded

For measurement of above ground shoot dry matter, air dried plant samples were sun dried and further dried in a hot air oven at 60° ± 2 °C, till constant weight was obtained in both rice and wheat. The biological yield was calculated by weighing the harvest of net plots. For determination of concentration of nitrogen (Kjeldahl’s apparatus), phosphorus (Vanado-molybdo-phosphoric acid yellow colour method, in nitric acid system), and potassium (flame photometer method) the procedure described by^69^ were followed. The concentration of zinc and iron was determined by using Atomic Absorption Spectrophotometer (AAS)^69^. The soil NaHCO_3_-extractable P was determined by Olsen’s method^70^; while soil 1 N ammonium acetate-extractable K was determined by flame photometric method. The DTPA-extractable Zn and Fe content in soil was determined by Atomic Absorption Spectrophotometer (AAS) as described by^71^. The microbial biomass carbon (MBC) was determined by fumigation method^72^ and soil chlorophyll was assayed using acetone: DMSO in ratio of 1:1 by using procedure given by^37^.

The system partial factor productivity (PFP) and agronomic efficiency (AE) for nitrogen and phosphorus was computed using the following expressions:

$$\mathrm{PFP}=\frac{\mathrm{YN}}{\mathrm{Na}}$$

$$\mathrm{AE}=\frac{\left(Yt-Yac\right)}{Na}$$

wherein, YN and Na refer to the cropping system grain yield (kg ha^−1^) and total nutrient (N or P) applied in cropping system (kg ha^−1^), Yt and Yac refer to cropping system grain yield (kg ha^−1^) in nutrient applied plots and in control plot (no nutrient), respectively.

### Data analysis

All the observations from the experiments were tabulated and observed to follow a normal distribution; hence the data was statistically analyzed using the F-test as per the procedure given by^73^. Least significant difference (LSD) values (*p* = 0.05) were used to determine the significance of difference between treatment means.
